# Risk Factors of Flatfoot in Children: A Systematic Review and Meta-Analysis

**DOI:** 10.3390/ijerph19148247

**Published:** 2022-07-06

**Authors:** Liya Xu, Hongyi Gu, Yimin Zhang, Tingting Sun, Jingjing Yu

**Affiliations:** 1 Faculty of Sports and Human Sciences, Beijing Sports University, Beijing 100084, China; 15207149098@163.com (L.X.); 13296523776@163.com (H.G.); 2 Key Laboratory of Sports and Physical Health Ministry of Education, China Institute of Sports and Health, Beijing Sports University, Beijing 100084, China; bsustt@163.com (T.S.); 13426236125@163.com (J.Y.)

**Keywords:** flatfoot, children, risk factors, meta-analysis

## Abstract

Background: This study aimed to explore the risk factors for flatfoot in children and adolescents to provide a reference basis for studying foot growth and development in children and adolescents. Methods: We examined the cross-sectional research literature regarding flatfoot in children and adolescents published in the past 20 years, from 2001 to 2021, in four electronic databases: PubMed, Web of Science, EBSCO, and Cochrane Library. Two researchers independently searched the literature according to the inclusion and exclusion criteria and evaluated the literature quality of the selected research; from this, a total of 20 articles were included in our review. After the relevant data were extracted, the data were reviewed using Manager 5.4 software (The Cochrane Collaboration, Copenhagen, Denmark), and the detection rate and risk factors for flatfoot in children were analyzed. Results: In total, 3602 children with flatfoot from 15 studies were included in the analysis. The meta-analysis results showed that being male (OR = 1.33, 95% CI: 1.09, 1.62, *p* = 0.005), being aged <9 years (age <6, OR = 3.11, 95% CI: 2.47, 3.90, *p* < 0.001; age 6–9 years, OR = 0.54, 95% CI: 0.41, 0.70, *p* < 0.001), joint relaxation (OR = 4.82, 95% CI: 1.19, 19.41, *p* = 0.03), wearing sports shoes (OR = 2.97, 95% CI: 1.46, 6.03, *p* = 0.003), being a child living in an urban environment (OR = 2.10, 95% CI: 1.66, 2.64, *p* < 0.001) and doing less exercise (OR = 0.25, 95% CI: 0.08, 0.80, *p* = 0.02) were risk factors for the detection of flatfoot. Conclusion: In summary, the detection rate of flatfoot in children in the past 20 years was found to be 25% through a meta-analysis. Among the children included, boys were more prone to flatfoot than girls, and the proportion of flatfoot decreased with age.

## 1. Introduction

Flatfoot is characterized by the collapse or over-flattening of the medial longitudinal arch of the foot [1]. Flatfoot in children is very commonly seen in clinical practice. Generally, it is physiological and manifests as flexible flatfoot, which is not a disease. In rare cases, flatfoot might also be pathological, manifesting as rigid flatfoot, such as in cases of congenital vertical talus, tarsal syndesmosis, etc. Most symptoms related to flatfoot gradually improve as a child gets older. Most previous studies have understood the formation of flatfoot to be related to the collapse of the medial longitudinal arch caused by abnormal bone structure in the foot or the relaxation of muscle ligaments [2]. However, in addition to physiological structure, many external factors affect the occurrence of flatfoot in children and adolescents [3]. Previous literature has shown that flatfoot in children causes parental anxiety due to concerns that some types of flatfoot cause fatigue or pain and development into pathological flatfoot [4]. Although a number of cross-sectional surveys have previously been conducted to study the relevant susceptibility factors for children, the sample sizes were small, the research results were uneven, and the accuracy of most studies due to the influence of various confounding factors remains to be discussed [5].

This study aimed to examine the literature published from 2001 to 2021 regarding the susceptibility factors for flatfoot in children and used a meta-analysis technique to evaluate the relevant literature and explore the susceptibility factors for flatfoot in children. This was conducted with the aim of forming an overall understanding and analyzing the results in combination with worldwide research to provide a reference basis for research regarding the growth and development of flatfoot in children and to determine directions for further research. The study will be useful in the formation of future clinical guidelines.

## 2. Materials and Methods

This systematic review and meta-analysis were performed following the Preferred Reporting Items for Systematic reviews and Meta-analysis (PRISMA) guidelines [6].

### 2.1. Study Search

A comprehensive search was conducted on PubMed, SCOPUS, Cochrane Library, and Web of Science from 1 January 2001 to 31 June 2021. The three main keywords were “flatfoot,” “child,” and “factor.” The synonym keywords were linked together by the “OR” operator for keywords within one factor and the “AND” operator for keywords between two elements. The first keywords were “flatfoot,” “pronated foot,” “pronated feet,” “pes planus,” “arch collapse,” “planovalgus,” “flat-arched feet,” “pes plano-valgus,” and “low arched feet.” The second keywords were “child,” “children,” “teenager,” “preschool child,” and “adolescent.” The factors keywords were “risk factors,” “influence factors,” and “related factors.”

### 2.2. Study Selection

The inclusion criteria were as follows: (i) studies concerning children who had flatfoot; (ii) studies with observational or cross-sectional designs; (iii) studies in which children with flatfoot and controls were diagnosed using a flatfoot assessment tool; (iv) studies reporting adequate data for pooling for the analysis; (v) studies published in English. The exclusion criteria were as follows: (i) review articles, letters, or comments; (ii) studies without available data for statistics.

### 2.3. Data Extraction

Two authors extracted all of the data from all of the eligible studies. Firstly, by reading the title and abstract of the literature, the literature that did not meet the inclusion criteria was excluded. The remaining pieces of literature were read and screened again to determine the literature that met the inclusion and exclusion criteria. The following variables were extracted from each study: the first author’s name, publication year, country, sample size, age, sex ratio, research factors, and study quality score. Any disagreement was resolved by discussion or consultation with a senior reviewer to reach a consensus. In addition, data extraction included reporting research results and their related statistical significance.

### 2.4. Quality Assessment

Two reviewers independently assessed the quality of the included studies using the cross-sectional research quality evaluation scale of the Agency for Health Care Research and Quality (AHRQ), which has 11 items in total. If the answer to the scale was “no” or “unclear,” 0 points were given; if the answer was “yes,” it was given 1 point. Documents with scores of 0–3 points were of low quality, documents with scores of 4–7 points were of medium quality, and documents with scores of 8–11 points were of high quality. Only studies with scores of 4 or more were included in the meta-analysis.

### 2.5. Data Analysis

Any disagreement was resolved by discussion or consultation with a senior reviewer to reach a consensus. Statistical analyses were undertaken using Review Manager 5.4 (The Cochrane Collaboration, Copenhagen, Denmark). The effect quantity was described using the risk factors for flatfoot in children and their 95% confidence intervals (Cis). We used the random-effects model or fixed-effects model according to the heterogeneity between the studies. We assessed statistical heterogeneity using the *I* square (*I*^2^) values (*I*^2^ > 50% was considered to imply statistical heterogeneity). The heterogeneity of the included literature was tested. When *p* ≥ 0.1 and *I*^2^ < 50%, there was no significant statistical heterogeneity, and the fixed-effects model was used; *p* < 0.1, *I*^2^ ≥ 50% indicated statistical heterogeneity, and in this case, the random-effects model was selected for combined analysis. Sensitivity analysis was carried out by comparing the differences between the fixed- and random-effects models, and publication bias was assessed using funnel plots.

## 3. Results

### 3.1. Search Results

**Figure 1:**
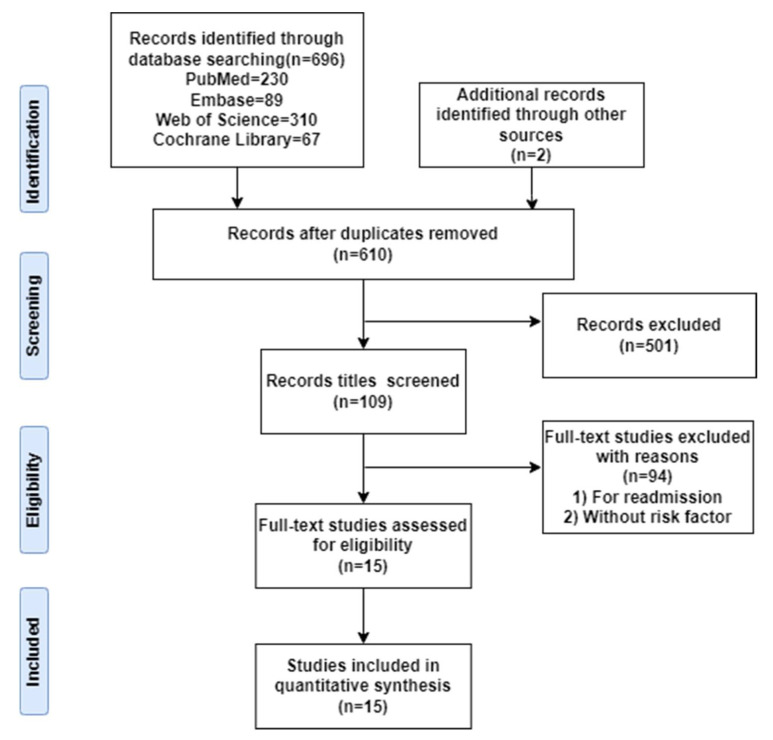
PRISMA flowchart.

Initially, we yielded 698 relevant studies from the electronic databases and 2 additional records through other sources (search through Google academic), of which 88 publications were excluded because they were duplications. After reading the title and abstract of these 610 papers, 501 papers were excluded as they did not fulfill the inclusion criteria. Ultimately, following the removal of duplicates and studies that did not meet the review’s inclusion criteria, 15 studies involving 14,483 participants were included. The flow diagram of study selection is shown in Figure 1.

### 3.2. Quality Assessment

A total of 3659 children with flatfoot symptoms were detected, and the total detection rate of flatfoot was 25.3%. The publication year of the 15 studies included [7,8,9,10,11,12,13,14,15,16,17,18,19,20,21] ranged from 2005 to 2021. Three studies were published in China, two studies originated from Greece, two originated from Australia, and the rest were from America, Spain, Nigeria, Italy, Poland, Japan, Ethiopia, and Mexico. The study quality scores ranged from 5 to 9. Among the 16 kinds of literature, 8 were medium-quality, and 7 were high-quality documents. After evaluating the study quality and the risk of bias, a total of eight HQ [7,8,11,12,13,14,18,20] studies and a further seven MQ [9,10,15,16,17,19,21] studies were identified. The extracted data relating to study characteristics are presented in Table 1.

### 3.3. Results of Meta-Analysis

According to the heterogeneity test results displayed in Table 2, the literature regarding children aged <9 years and regional factors was analyzed using a fixed-effects model to combine the effect quantities. The literature regarding age 9–12 years, sex, BMI (body mass index), joint relaxation, shoe type, school type, and exercise level was analyzed using a random-effects model to combine the effect quantities.

### 3.4. Risk Factors

The meta-analysis results showed that being male, being aged <9 years, joint relaxation, wearing sports shoes, being a child living in an urban environment, and doing less exercise were the risk factors for the detection of flatfoot.

**Figure 2:**
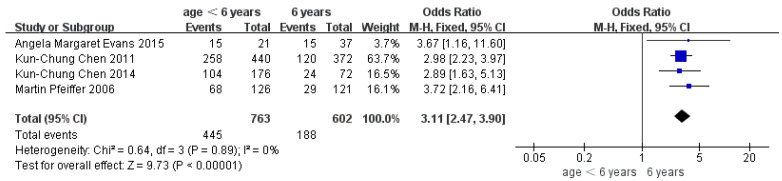
The forest plot of age <6 years for flatfoot in children [8,12,14,17].

**Figure 3:**
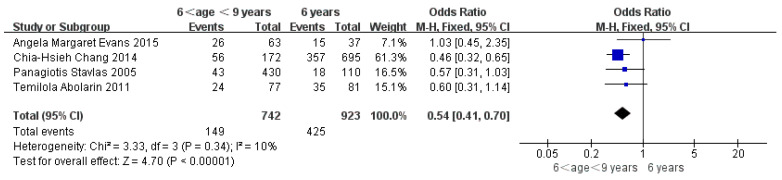
The forest plot of age 6–9 years for flatfoot in children [7,11,13,17].

**Figure 4:**
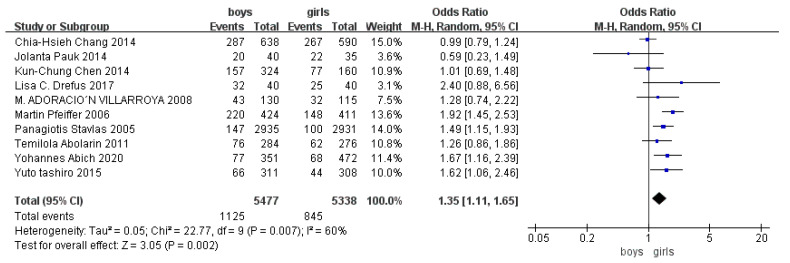
The forest plot of sex for flatfoot in children [7,8,9,11,13,14,16,18,19,20].

**Figure 5:**
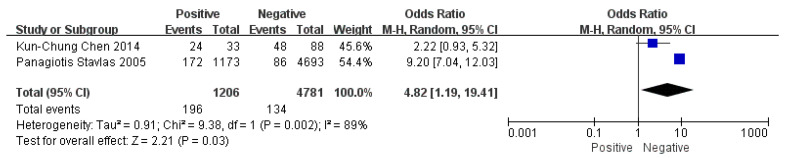
The forest plot of joint relaxation for flatfoot in children [7,14].

**Figure 6:**
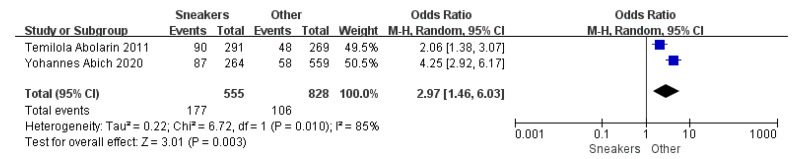
The forest plot of shoe shape for flatfoot in children [11,20].

**Figure 7:**
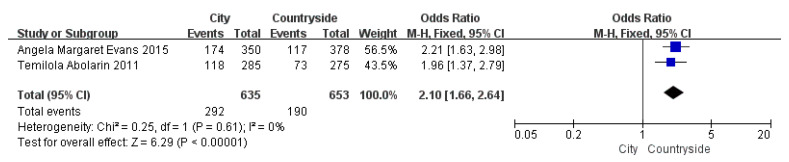
The forest plot of the region for flatfoot in children [11,17].

**Figure 8:**
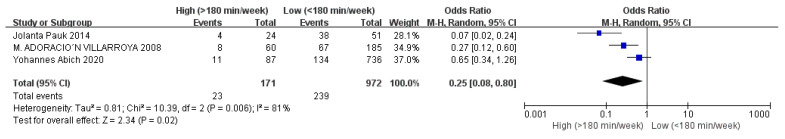
The forest plot of exercise time for flatfoot in children [9,16,20].

**Figure 9:**
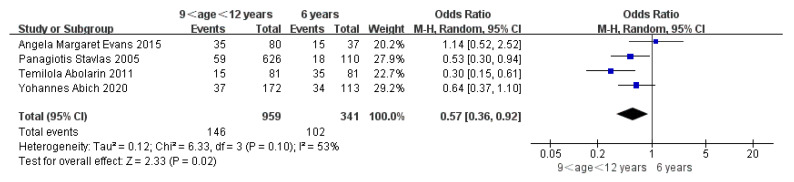
The forest plot of age 9–12 years for flatfoot in children [7,11,17,20].

**Figure 10:**
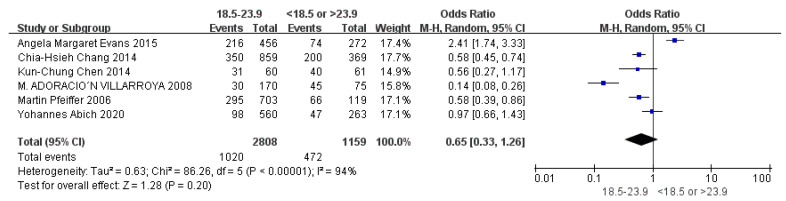
The forest plot of BMI for flatfoot in children [8,9,13,14,17,20].

Children who had flatfoot were significantly younger (age < 6, OR = 3.11, 95% CI: 2.47, 3.90, *p* < 0.001, Figure 2; age 6–9 years, OR = 0.54, 95% CI: 0.41, 0.70, *p* < 0.001, Figure 3). There is strong evidence from nine studies that sex was also associated with flatfoot in univariable analyses or meta-analyses. In terms of sex, boys were more likely to suffer from flatfoot than girls (boys, OR = 1.35, 95% CI: 1.11, 1.65, *p* = 0.002, Figure 4). Joint relaxation (OR = 4.82, 95% CI: 1.19, 19.41, *p* = 0.03, Figure 5), wearing sports shoes (OR = 2.97, 95% CI: 1.46, 6.03, *p* = 0.003, Figure 6), being a child in living an urban environment (OR = 2.10, 95% CI: 1.66, 2.64, *p* < 0.001, Figure 7), and doing less exercise (OR = 0.25, 95% CI: 0.08, 0.80, *p* = 0.02, Figure 8) were also risk factors for flatfoot. At the same time, the protective factors for flatfoot in children and adolescents were age 9–12 years (OR = 0.57, 95% CI: 0.36, 0.92, *p* = 0.02, Figure 9), a lower BMI (18.5 < BMI < 23.9, OR = 0.65, 95% CI: 0.33, 1.26, *p* = 0.20, Figure 10), being a child living in a countryside environment, and doing more exercise.

### 3.5. Sensitivity Analysis

The fixed- and random-effects models were used for sensitivity analysis for the nine factors included. It can be seen from Table 3 that the OR value (95% CI) results of the two effect models were relatively close, and the *I*^2^ value was the same. On the surface, the meta-analysis of this study was stable.

### 3.6. Publication Bias Assessment

**Figure 11:**
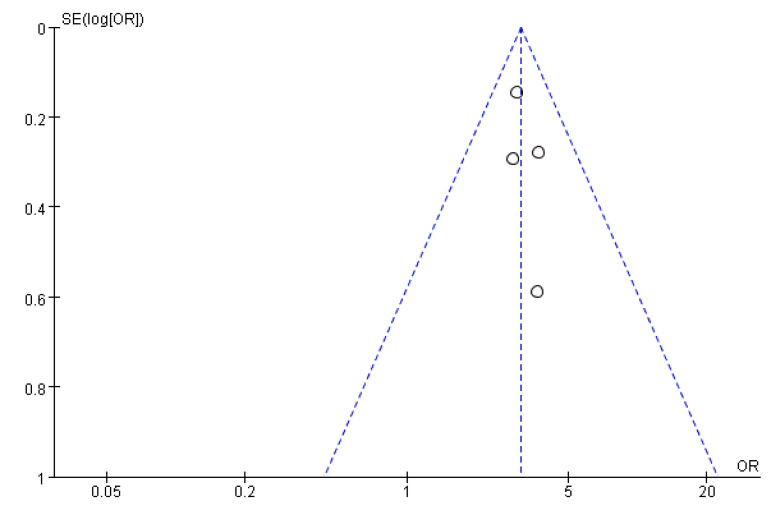
The funnel plot of publication bias for age <6 years for flatfoot in children.

**Figure 12:**
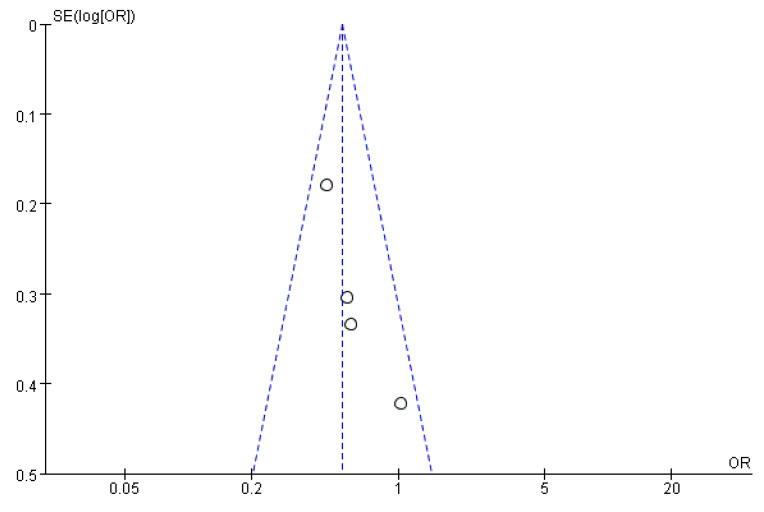
The funnel plot of publication bias for age 6–9 years for flatfoot in children.

**Figure 13:**
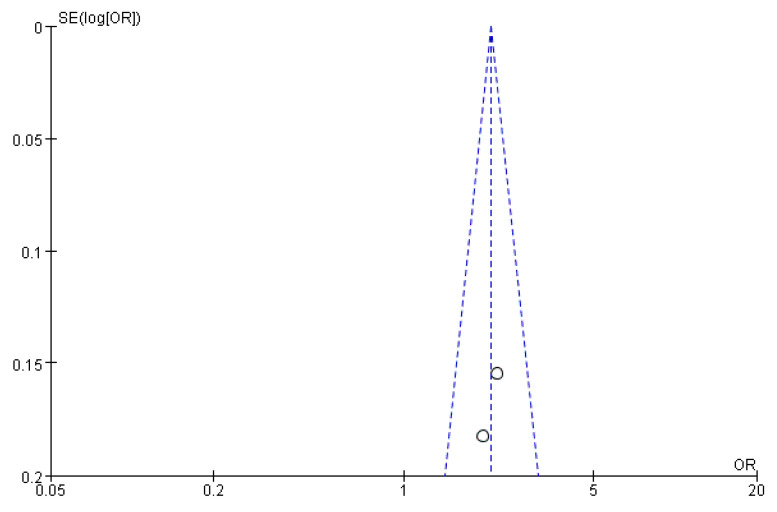
The funnel plot of publication bias for the region for flatfoot in children.

Figure 11, Figure 12 and Figure 13 show that the points of the funnel diagram for the factors of age < 9 years and region were almost perfectly evenly distributed on both sides of the axis, indicating that there is a lower possibility of publication bias in the literature included in this study and that the results are more reliable. The points of the funnel map for other factors were unevenly distributed on both sides of the axis, indicating a high possibility of publication bias in the literature included in this study, which might be due to the small number of articles in the literature.

## 4. Discussion

Flatfoot was divided into two types: congenital and acquired. The children included in this analysis were generally born with mild dorsiflexion and valgus. Almost all of the infants were flatfooted due to immature physiological development. With gradual growth and development, plantar fat gradually disappeared, the valgus was reduced, and the longitudinal and transverse arches of the foot began to be significant [22]. Acquired flatfoot was mainly affected by external factors, such as physical activity level, shoe-wearing habits, and living area. Adolescents are in a critical period of growth and development, and the preschool age is the main stage of foot arch development [23]. If an abnormal arch persists or complications such as pain and bone deformity continue, this will seriously affect a child’s health-related quality of life. There were some differences in the epidemiological incidence rate of flatfoot, but it is believed that flatfoot in children improves with age. This study collated the relevant literature published in recent years to explore the susceptible factors for flatfoot in children. We provided a reference basis for studying foot growth and development in children.

The detection rate of flatfoot in girls and boys was different. Compared with girls, the detection rate of flatfoot in boys was higher. This was consistent with research results presented by most scholars. Panagiotis [7] showed that the detection rate of flatfoot was 5.0% for boys and 3.4% for girls; Martin’s [8] result showed that the detection rate of flatfoot was 52% for boys and 36% for girls. Other scholars have concluded that the risk of flatfoot in men is always higher than that in women, and the risk is not significantly related to age. This may be related to the fact that growth and development take place earlier in girls than boys. The development of posture balance and physical development also takes place earlier in girls. The physiological process of the growth of foot arches from low arches to normal arches occurs earlier in girls, the development of boys’ medial longitudinal arches is slower than girls’, and boys’ plantar fat pads are thicker than girls’. However, it needs to be determined whether every age group conforms to these norms because women’s faster growth and development may be affected by body fat rate. For example, a study in Nigeria found there is a higher incidence of flatfoot at an older age in women [24]. Due to the fact that the studies took place using different countries, regions, and age ranges, it was impossible to make direct comparative analyses. Nevertheless, as age increases, some sex differences tend to remain stable.

The detection rates for flatfoot in children and adolescents were different in different age groups. The results of this study showed that the detection rate of flatfoot tends to decrease with the increase in age. Panagiotis [7] found that the proportion of high and low arches decreased with age in both men and women. Martin Pfeiffer [8] found that flatfoot was detected in 54% of children in the 3-year-old group, while flatfoot was only detected in 24% of children in the 6-year-old group. In a cross-sectional survey conducted in 2020, Yohanes [20] also found that the younger the age, the greater the probability of detecting flatfoot. Some studies also showed that the incidence of flatfoot in children and adolescents showed a downward trend from 72.6% to 37.9% at the age of 7–12 [25]. It was proposed that the foot arch of Chinese children is still in the process of development at the age of 7–12. Therefore, we can speculate that if all of the subjects were of preschool age, the incidence of flatfoot would increase. With the increase in age, the incidence of flatfoot would gradually decline, which would also agree with the physiological development norms seen in foot arches. However, the development of the foot arch might not be a continuous development process, similar to the growth patterns of height or weight. The foot structure may change when children develop new motor skills or ambulation. This change may occur suddenly at a particular time point without fixed rules. A prospective cohort study could be conducted to verify this idea.

Joint ligament relaxation was also a critical factor in the occurrence of flatfoot in children and adolescents. Most studies showed that the incidence of joint relaxation was higher in girls than in boys [26]. This may be related to girls’ joint flexibility being more significant than that of boys. Children undergo a period of joint ligament development. During this stage, joint ligament relaxation may be caused by underdevelopment. It is a self-construction and self-organization process in the human body and is the embodiment of the body’s gradual maturity. Generally, joint ligament relaxation gradually improves with age. Most occurrences are due to everyday physiological phenomena. However, although the range of joint activity in girls is better than that in boys, there is a dominant gender difference. Generally speaking, boys have a greater likelihood of having flatfoot than girls. Therefore, the range of joint motion could be analyzed as a gender-confounding factor.

In children living in a city or in studies in which children were recruited from an urban area, urban area was also a susceptible factor for flatfoot in children. In 2011, Temilola [11] investigated the incidence and related predictors of flatfoot among school-age children in urban and rural areas in southwestern Nigeria. The study found that more than half of urban children (51.2%) had flatfoot, compared with 35% of rural children. It was found that this may be related to children’s lifestyles and eating habits in urban areas. Some studies have found that the vast majority of children in cities (more than 90%) wear closed shoes, while most children in rural areas (69.5%) wear sandals [11,27], and wearing closed shoes affects the development of the longitudinal arch more than sandals or sandals, which indirectly led to a higher detection rate of flatfoot in urban children than that in rural children [28]. Therefore, the effect of shoe type could be used as a confounding factor for residences. In addition, the overweight and obesity rates in urban children were higher than those in rural children due to their high living standards, which may also be a susceptibility factor that leads to a high detection rate of flatfoot.

In terms of physical activity, children who exercised less were more likely to have flatfoot. In some studies, some children had flatfoot due to low levels of physical activity [20]. Low levels of physical activity could lead to delayed or uneven muscle strength, resulting in poor arch strength. The exercise was closely related to physical development, weight management, and a healthy lifestyle [29]. However, children need to engage in appropriate physical activities. Adolescents who are not fully developed should avoid taking part in overloaded labor (such as burden-bearing) and sports (such as weight lifting). They could engage in high leg lifting, jumping activities (such as rope skipping, long jump, high jump, vertical take-off, etc.), and climbing activities (such as climbing ladders, using balance beams, rope climbing, pole climbing, etc.) to fully exercise the muscles and ligaments of the arch of the foot.

In this study, BMI was not a relevant influencing factor of flatfoot in children, which may have been caused by insufficient sample sizes or the different countries and regions included in the study population. However, previous studies reported the relationship between BMI and flatfoot. In a 2020 survey on flatfoot-related factors regarding 823 children and adolescents aged 11–15 in Ethiopia, Yohanes found that the detection rate of flatfoot in children with low weights was lower than that in children with average weights [20]. However, a previous study regarding flatfoot in preschool children found that children with low weights had a two times higher risk of flatfoot than children with average weights. Therefore, these two reports show the importance of the developmental stage in the incidence of flatfoot. Adoracio [9] found that obesity had a significant impact on the foot structure of children, and the detection rate of flatfoot was higher in obese children. Martin [8] showed that the incidence of flatfoot in overweight and obese children was three times higher than that in average-weight children. Dowling [30] studied plantar pressure distribution in obese children. They concluded that sustained excessive weight in a child seems to flatten the foot arch during walking, and these children would also have a significantly increased risk of foot diseases. Taylor [31] studied orthopedic complications in overweight children and showed that overweight children more commonly had movement disorders, which led them to avoid sports activities. In time, this led to weight gain and an increase in the incidence of flatfoot. However, the difference in foot structures between overweight or obese children with average-weight children did not mean that all overweight or obese children had flatfoot. In a study in 2015, Angela [17] put forward a critical view that the relationship between relevant factors cannot be simply unified as causality. Although most studies determined that flatfoot was more likely to occur in heavier children, it cannot be assumed that there was a specific link between the foot arch and BMI in children. This study suggested that there was no specific relationship between the increase in BMI in children and the occurrence of flatfoot, which conflicted with the results of many other studies [32].

The methods of evaluating and measuring flatfoot mainly include plantar pressure tests, X-rays, and the foot posture index (FPI), which are useful tools for evaluating flatfoot in children. The diagnosis of flatfoot is not difficult. A traditional and simple method for the measurement of flatfoot is footprint analysis. With the progress of science and technology, advanced instruments might be gradually popularized, and plantar pressure testing will be more widely used in biomechanics. In a large sample survey, a 3D laser scanner was applied to conduct rapid clinical screening and post-treatment effect evaluation. The best time to correct flatfoot is 3–12 years old because flatfoot is not easy to see when children are very young, especially at the age of one to two years old, when the child’s foot arch has not been formed, and at this age, children also walk unsteadily, so it is difficult to determine the presence of abnormalities. As children age, their left and right foot arches gradually form, and their feet become increasingly stable and powerful. When they reach the age of 12, they develop and take shape. If the best treatment time is not taken advantage of, a child’s feet will be fully developed, and they may develop stiff flatfoot. Therefore, the division of age in this study also referred to the best age range for flatfoot correction.

The healthy development of the foot arch is significant in the critical period in the growth and development of children [33]. Proper physical activity aids the development of foot arches and reduces the incidence of flatfoot. Parents should choose suitable shoes for their children and help their children control their weight within the average range during the critical period of foot arch development. According to the results of this study, which focused on the foot arch development of children, we should pay attention to both internal and external factors, encourage children to engage in appropriate exercises, including jumping and other activities, to promote foot development, increase children’s opportunity to walk barefoot, and avoid excessive weight-bearing and sitting for long periods of time. However, with regard to physical exercise, activities that have a substantial impact on the foot’s arch should be avoided to prevent foot injuries. In some children with symptomatic flatfoot, appropriate arch pads can also be used to adjust the plantar pressure distribution [34].

Although this study strictly followed the inclusion and exclusion criteria to screen the relevant literature for meta-analysis, there were still some limitations. The sample sizes in the original research literature used in this study were quite different, and the research assumptions and analysis methods included in the study were also different, which had a particular impact on the research results. All of the literature included in this study were cross-sectional studies that mainly compared and analyzed the detection rate and related factors of flatfoot in children, but they did not include prospective cohort studies and case–control studies. The investigation and analysis of related factors were not comprehensive enough. The occurrence of flatfoot was shown to be affected by internal factors (age, sex, nutritional status, genetics, race, and development differences) and other external factors (shoe shape, environmental conditions, and physical activities). The distinct limitation of this study design is that no separate the external and internal factors affecting flatfoot, which is likely to be a significant confounder, as more external and internal factors progressions increase the risk of flatfoot development.

## 5. Conclusions

To summarize, through a meta-analysis, we found that the detection rate of flatfoot in children was 25% in the past 20 years. Among the children included in the studies, boys were more prone to flatfoot than girls, and the proportion of flatfoot decreased with age.

## Figures and Tables

**Table 1:** The general characteristics of the studies included.

Study	Country	Age (Year)	Sample Size	Detection Rate (%)	Research Factors	Measuring Method	AHRQ Score
Panagiotis 2005 [7]	Greece	6–17	5866	4.4	Sex, age, joint relaxation	the CSI, the AI	H
Martin 2006 [8]	Greece	3–6	835	44	Sex, age, BMI	the RA	H
Villarroya 2008 [9]	Spain	9–17	245	30.6	Sex, BMI, exercise level	the FPI	MD
Twomey 2010 [10]	Australia	9–12	52	51.9	Sex	the HFMM	MD
Temilola 2011 [11]	Nigeria	6–12	560	24.7	Sex, age, shoe type, region	the FPI	H
Chen 2011 [12]	China	3–6	2638	44	Age	the CA, the CSI, the AI	H
Chang 2014 [13]	China	6–10	1228	45.3	Sex, age, BMI	the FPI	H
Chen 2014 [14]	China	3–6	484	48.5	Sex, age, BMI, joint relaxation, and movement time	the CSI	H
Galli 2014 [15]	Italy	9.6	140	88	Joint relaxation	the AI	MD
Jolanta 2014 [16]	Poland	9–16	75	56	Sex, exercise time, school type	the CA	MD
Angela 2015 [17]	Australia	3–15	728	40	Age, BMI, region	the FPI	MD
Yuto 2015 [18]	Japan	10–12	619	17.8	Sex, age	the FPI, the TGS	H
Lisa 2017 [19]	America	6–12	60	55	Sex	the AHI	MD
Yohannes 2020 [20]	Ethiopia	11–15	823	17.6	Sex, age, school type, BMI, shoe type, exercise time	the FPI, the LLAS	H
Anna 2021 [21]	Mexico	5–9	50	57.7	Age, BMI	the CA	MD

AHRQ, Agency for Health Care Research and Quality; BMI, body mass index; H, high; MD, moderate; CSI, Chippaux–Smirak index; AI, arch index; RA, rearfoot angle; FPI, foot posture index; HFMM, Heidelberg foot measurement method; CA, Clarke’s angle; TGS, toe grip strength; AHI, arch height index; LLAS, lower limb hypermobility score.

**Table 2:** Meta-analysis of risk factors for flatfoot in children.

Risk Factor		Studies	Statistically Methods	OR with 95% CI	*I*^2^ (%)	*p*-Value
Age	Age < 6 years	4	Fixed	3.11 [2.47, 3.90]	0	*p* < 0.001
	age 6–9 years	4	Fixed	0.54 [0.41, 0.70]	10	*p* < 0.001
	age 9–12 years	4	Random	0.57 [0.36, 0.92]	53	*p* = 0.02
Sex	Boy	9	Random	1.35 [1.11, 1.65]	60	*p* = 0.002
	Girl					
BMI	18.5–23.9	6	Random	0.65 [0.33, 1.26]	94	*p* = 0.20
	<18.5 or >23.9					
Joint relaxation	Positive	2	Random	4.82 [1.19, 19.41]	89	*p* = 0.03
	Negative					
Shoe shape	Sneakers	2	Random	2.97 [1.46, 6.03]	85	*p* = 0.003
	Other					
Region	City	2	Fixed	2.10 [1.66, 2.64]	0	*p* < 0.001
	Countryside					
School type	Public	2	Random	0.27 [0.06, 1.37]	87	*p* = 0.11
	Private					
Exercise time	Long exercise time (>180 min/week)	3	Random	0.25 [0.08, 0.80]	81	*p* = 0.02
	Short exercise time (<180 min/week)					

BMI, body mass index.

**Table 3:** Sensitivity analysis of related factors for flatfoot in children.

Related Factors	Fixed-Effects Model		Random-Effects Model	
	*I* ^2^	OR (95% CI)	*I* ^2^	OR (95% CI)
Age < 6 years	0	3.11 [2.47, 3.90]	0	3.11 [2.47, 3.90]
age 6–9 years	10	0.54 [0.41, 0.70]	10	0.55 [0.41, 0.73]
age 9–12 years	53	0.57 [0.42, 0.78]	53	0.57 [0.36, 0.92]
Sex	63	1.34 [1.20, 1.50]	63	1.35 [1.11, 1.65]
BMI	94	0.80 [0.69, 0.92]	94	0.65 [0.33, 1.26]
Joint relaxation	89	7.84 [6.05, 10.16]	89	4.82 [1.19, 19.41]
Shoe shape	85	2.98 [2.27, 3.91]	85	2.97 [1.46, 6.03]
Region	0	2.10 [1.66, 2.64]	0	2.10 [1.67, 2.64]
Exercise time	81	0.34 [0.21, 0.55]	81	0.25 [0.08, 0.80]

## Data Availability

The data presented in this study are openly available in the studies referenced in the figures. The individual data in each can be seen in the original manuscripts.
